# Supplementary material for: Effects of Essential Oil Fumigation on Potato Sprouting at Room-Temperature Storage
Source: Plants (Basel). 2022 Nov 15;11(22):3109. doi: 10.3390/plants11223109 (PMC9695867; doi:10.3390/plants11223109)
Supplement: Supplementary file 1 [file plants-11-03109-s001.zip › plants-2013022-supplementary.pdf]

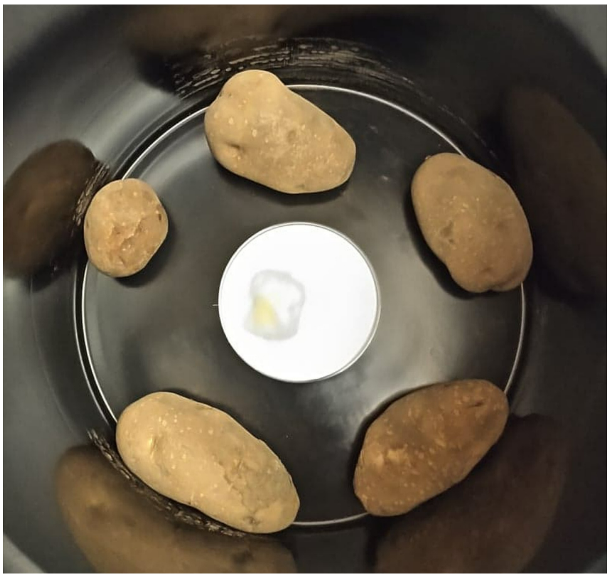

(a)

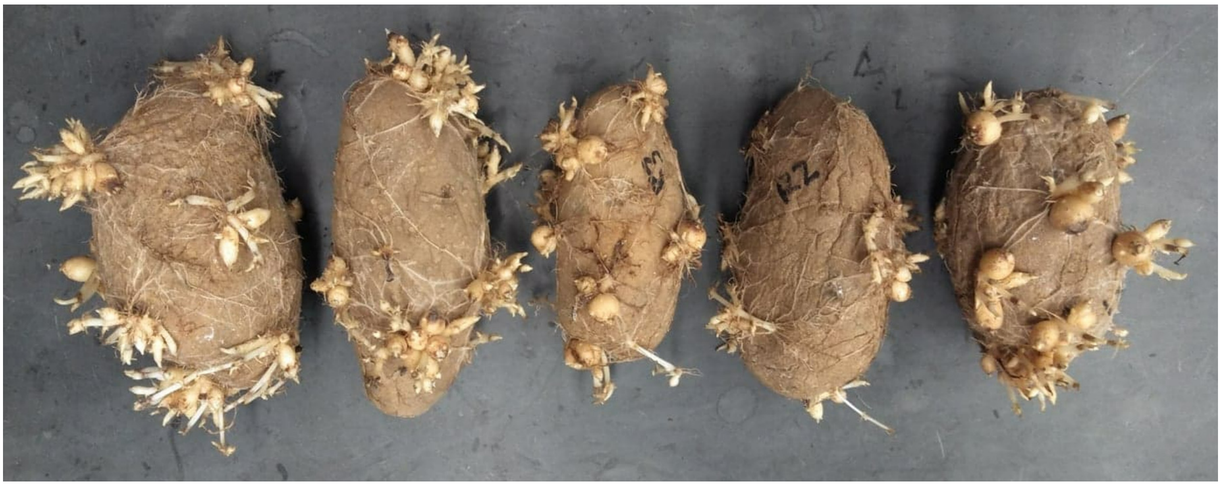

(b)

Supplementary Figure S1. Potato tubers treated with **(a)** *A. herba-alba* EO and **(b)** distilled water at 60 days of storage.
